# *Levilactobacillus brevis*, autochthonous to cucumber fermentation, is unable to utilize citric acid and encodes for a putative 1,2-propanediol utilization microcompartment

**DOI:** 10.3389/fmicb.2023.1210190

**Published:** 2023-07-26

**Authors:** Ilenys M. Pérez-Díaz, Clinton A. Page, Lesley Mendez-Sandoval, Suzanne D. Johanningsmeier

**Affiliations:** USDA-Agricultural Research Service, Food Science Research Unit, Raleigh, NC, United States

**Keywords:** cucumber fermentation, *Levilactobacillus brevis*, rep-PCR-(GTG)_5_, phenotyping, comparative genomic

## Abstract

The metabolic versatility of *Levilactobacillus brevis*, a heterofermentative lactic acid bacterium, could benefit environmentally compatible and low salt cucumber fermentation. The biodiversity of *Lvb. brevis* autochthonous to cucumber fermentation was studied using genotypic and phenotypic analyses to identify unique adjunct cultures. A group of 131 isolates autochthonous to industrial fermentations was screened using rep-PCR-(GTG)_5_ and a fermentation ability assay under varied combinations of salt (0 or 6%), initial pH (4.0 or 5.2), and temperature (15 or 30°C). No apparent similarities were observed among the seven and nine clusters in the genotypic and phenotypic dendrograms, respectively. A total of 14 isolates representing the observed biodiversity were subjected to comparative genome analysis. The autochthonous *Lvb. brevis* clustered apart from allochthonous isolates, as their genomes lack templates for citrate lyase, several putative hypothetical proteins, and some plasmid- and phage-associated proteins. Four and two representative autochthonous and allochthonous *Lvb. brevis*, respectively, were subjected to phenotype microarray analysis using an Omnilog. Growth of all *Lvb. brevis* strains was supported to various levels by glucose, fructose, gentiobiose, 1,2-propanediol, and propionic acid, whereas the allochthonous isolate ATCC14890 was unique in utilizing citric acid. All the *Lvb. brevis* genomes encode for 1,2-propanediol utilization microcompartments. This study identified a unique *Lvb. brevis* strain, autochthonous to cucumber, as a potential functional adjunct culture for commercial fermentation that is distinct in metabolic activities from allochthonous isolates of the same species.

## Highlights

- 131 *Lvb. brevis* from cucumber generated 7 rep-PCR-(GTG)_5_ clusters.
- 99 *Lvb. brevis* from cucumber generated 9 fermentation ability clusters.
- 14 biodiverse species of *Lvb. brevis* comprise a unique subclade.
- *Lvb. brevis* from cucumber lacks citrate lyase.
- The species encodes for putative Pdu microcompartments.

## 1. Introduction

*Levilactobacillus brevis*, a heterofermentative lactic acid bacterium, co-exists with *Lactiplantibacillus pentosus* in commercial-scale cucumber fermentations brined with 1.06 M (6%) sodium chloride (NaCl) and is usually detected from days 7 to 14 post-tanking (Pérez-Díaz et al., 2017). Cucumber fermentations that host *Lvb. brevis* were found to suffer from bloater defect, the formation of hollow cavities inside the fruits, as the result of an increment in internal pressure by carbon dioxide production (CO_2_) (Etchells et al., 1968; Zhai et al., 2018). The ability of *Lvb. brevis* to produce carbon dioxide from the heterofermentation of glucose and fructose prompted such an association (Etchells et al., 1968; Zhai et al., 2018). However, more recent studies indicate that bloater defects develop in cucumber fermentation well before *Lvb. brevis* reaches maximum densities and can occur in its absence (Zhai and Pérez-Díaz, 2020). Understanding that *Lvb. brevis* is not the primary contributor to the CO_2_-mediated bloater defect creates opportunities to exploit its metabolic versatility as an adjunct or starter culture for cucumber fermentation given its competitiveness in such a habitat.

The diversion of a portion of the carbon from sugars to acetic acid *via* heterofermentation instead of solely lactic acid formation from homofermentation may present technological advantages for the retention of cucumber tissue firmness and enhancement of flavor in pickling. For instance, pure cultures of the heterofermentative lactic acid bacteria *Lvb. brevis* produced less acid and firmer fermented olives (Etchells et al., 1974; Portilha-Cunha et al., 2020). Moreover, desirable flavors form in fermented vegetables from the combination of volatile compounds derived from the vegetable and those that are naturally produced by microbes. To date, more than 200 volatile compounds have been identified in fermenting cucumber or commercial tankyard brines (Zhou and McFeeters, 1998; Marsili and Miller, 2000; Johanningsmeier and McFeeters, 2011), but the relative contributions of fermentation microbiota are not known. Early work showed that cucumber fermented with pure cultures of *Lvb. brevis* was perceived as more aromatic and less acidic than those fermented with *Lpb. plantarum*, which were more acidic, less bitter, and had more raw cucumber flavor (Aurand et al., 1965). None of the pure cultures tested could independently reproduce the flavor profile of the indigenous ferments, and there was significant strain variation within each species (Aurand et al., 1965). Considering such findings, it is relevant to investigate the indigenous diversity of heterofermentative lactic acid bacteria such as *Lvb. brevis* in industrial-scale cucumber fermentation.

Inclusion of *Lvb. brevis* in starter cultures for vegetable fermentation may also contribute to greater long-term microbial stability. Utilization of a tripartite starter culture of *Lpb. pentosus, Lvb. brevis*, and *Lentilactobacillus buchneri* in Cucumber Juice Medium (CJM) resulted in microbial stability at 60 days of storage (Ucar et al., 2020a). *Lvb. brevis* was found to utilize xylose and trehalose in CJM (Ucar et al., 2020a). Both these sugars are intrinsic to cucumber fermentation (Ucar et al., 2020a) and are depleted during anaerobic spoilage along with other trace mono- and disaccharides (Johanningsmeier and McFeeters, 2015). *Lvb. brevis* was also found to utilize the cellulose-derived disaccharide gentiobiose in CJM, which may be relevant should this sugar become available in cucumber fermentation (Ucar et al., 2020b). The putative ability of *Lvb. brevis* to utilize xylose, trehalose, and gentiobiose is hypothesized to aid in precluding spoilage microbes from deriving energy for growth in cucumber fermentation, thus conferring microbial stability.

While *Lvb. brevis* spontaneously co-dominates in commercial cucumber fermentations brined with 6% sodium chloride (Pérez-Díaz et al., 2017), most members of this species are sensitive to infection by indigenous bacteriophages (Lu et al., 2012). It is presumed that, given the sensitivity to bacteriophages and the fact that *Lvb. brevis* is a slow grower in cucumber fermentations compared to *Lpb. plantarum* and *Lpb. pentosus*, only a fraction of the acetic acid and carbon dioxide formed are contributed by such a bacterium (Pérez-Díaz et al., 2017).

Lack of an understanding of the intrinsic biodiversity of *Lvb. brevis* in commercial cucumber fermentation impedes the identification of adjunct starter cultures that may aid in enhancing quality, flavor, and microbial stability. Thus, this study was designed to screen the intrinsic biodiversity of *Lvb. brevis* autochthonous to commercial fermentations to enable the selection of genetically and phenotypically unique strains as adjunct starter culture candidates. A collection of 131 *Lvb. brevis* isolated from commercial fermentations in the seasons of 2010 in North Carolina and 2012 in Minnesota (Pérez-Díaz et al., 2017) was analyzed to define genetic biodiversity using rep-PCR-(GTG)_5_. The screening of the ability of 99 isolates from North Carolina to ferment cucumber juice was done under varied conditions of pH, temperature, and salt concentration. The genome sequences of representative *Lvb. brevis* were subjected to comparative genome analysis to understand putative biodiversity among isolates from a common habitat relative to allochthonous strains. Four out of the fourteen *Lvb. brevis* isolates selected for comparative genome analysis that represent unique phylogenetic clusters were used for phenotype microarrays using an Omnilog system to study carbohydrate utilization.

## 2. Materials and methods

### 2.1. *Lvb. brevis* collection and culture conditions

The 131 *Lvb. brevis* included in this study were isolated and identified as described by Pérez-Díaz et al. (2017) from commercial fermentations in North Carolina (99) and Minnesota (32). All 131 *Lvb. brevis* are maintained in the USDA-ARS Food Science and Market Quality & Handling Research Unit (Raleigh, NC) culture collection as frozen stocks prepared in Lactobacilli MRS broth supplemented with 15% glycerol (Pérez-Díaz et al., 2017). The cultures were transferred from frozen stocks to Lactobacilli MRS broth prior to inoculating the experimental media described below. Cultures were incubated at 21 ± 1°C for 48 h under anaerobic conditions. We have observed that *Lvb. brevis*, autochthonous to cucumber fermentation, grows faster at ambient temperature (21 ± 1°C) than at 37°C.

### 2.2. Genotyping of the *Lvb. brevis* isolates using Rep-PCR-(GTG)_5_

DNA was extracted from a pure culture of *Lvb. brevis* using the MasterPure™ DNA purification kit (Cat No.: MCD85201, Epicenter, Madison, WI, USA) according to the manufacturer's instructions. The extracted DNA was stored at −20°C until further use. Rep-PCR-(GTG)_5_ was performed following the method by Versalovic et al. (1994) with the modifications described by Pérez-Díaz et al. (2021). Amplicons were analyzed as described by Pérez-Díaz et al. (2021) using the Pearson correlation coefficient in Bionumerics version 7.6.3 (Applied Math, Belgium) to build similarity matrices of the densitometric curves and the unweighted pair group method with arithmetic averages (UPGMA) for clustering.

### 2.3. Phenotyping of the *Lvb. brevis* isolates using a fermentation ability screening design

Pure cultures were subjected to a CJM fermentation ability screening using a fractional factorial design as described by Pérez-Díaz et al. (2021). Briefly, four treatments were used with variable initial pH, NaCl concentration, and incubation temperature, as follows: treatment 1: initial pH 5.4, 0% NaCl, and incubation at 15°C; treatment 2; initial pH 4.0, 6% NaCl, and incubation at 15°C; treatment 3: initial pH 5.4, 6% NaCl, and incubation at 30°C; and treatment 4: initial pH 4.0, 0% NaCl, and incubation at 30°C. The CJM was prepared as described by Pérez-Díaz et al. (2021). CJM pH was measured after 48 h of fermentation as an indicator of each isolate's ability to rapidly initiate fermentation under a variety of environmental conditions. Two-way hierarchical clustering of the mean fermentation pH values of each isolate under the four fermentation conditions was performed, and a screening analysis was conducted to determine the variables that most impacted the fermentation ability of the *Lvb. brevis* isolates (JMP version 10.0, SAS Institute, Cary, NC).

### 2.4. Comparative analysis of fourteen *Lvb. brevis* genomes

The sequencing, assembly, and annotation of the 14 *Lvb. brevis* genomic DNA sequences included in this study (30.8.38, 30.2.29, 7.8.43, 7.8.34, 7.8.33, 7.2.13, 7.2.49, 14.2.24, 3.2.41, 3.8.25, 7.2.12, 14.2.10, 7.2.41, and 7.2.40) are described by Page and Pérez-Díaz (2021) and Page et al. (2022). Briefly, PATRIC (Davis et al., 2020) was used for the initial assembly and annotation of the DNA sequences; Unicycler version 0.4.8 (Wick et al., 2017) was used for *de novo* assemblies; and RASTtk (Brettin et al., 2015) was used for annotation. Genbank accession numbers and SRA locators for the 14 *Lvb. brevis* genomic DNA sequences are found in Supplementary Table 1. Phylogenetic trees were constructed using PATRIC/BV-VRC version 3.26.4 (Wattam et al., 2017) *via* the Codon Tree method, which employs RaxML to generate phylogenetic distances between sequences (Stamatakis, 2014). A total of 477 open reading frames were used for the comparison, with *Lpb. pentosus* strain LA0445 included as an outgroup to root the tree. Tree diagrams were generated with Interactive Tree of Life (iTOL) v. 6.5.7 (Letunic and Bork, 2021). Default parameters were applied when using both bioinformatic tools.

### 2.5. Comparative analysis of the putative and deduced proteins encoded by the *L. brevis* genomes and protein family sorting

Of 14, four *Lvb. brevis* genome sequences representing each of the three branches in the phylogenetic tree for autochthonous isolates were subjected to a comparative analysis of the putative and deduced proteins and a protein family sorting using PATRIC-BV-VRC-version 3.26.4 (Wattam et al., 2017). The comparative analysis was conducted using the bidirectional BLASTP service. The deduced protein sequences from the four autochthonous *Lvb. brevis* isolates, including 30.2.29, 3.2.41, 7.8.43, and 14.2.10 were compared against *Lvb. brevis* ATCC14869, the type strain for the species, isolated from human feces. Another three *Lvb. brevis* genome sequences were added to the comparison, including SA-C12, YSJ3, and NPS-QW-145, which were isolated from food fermentations. GenBank accession numbers for all the genome sequences used are listed in Supplementary Table 1. The tabulated results for the putative and deduced protein profile comparison can be found in Supplementary Table 2. Genes of interest due to their absence or presence in allochthonous or autochthonous isolates were further evaluated using the Compare Region Viewer tool in PATRIC-BV-VRC equipped with cross-genus families (PGfams) comparison. For the protein family sorting, we used *Lvb. brevis* NPS-QW-145, SA-C12, and YSJ3 as reference strains. The results generated by the protein sorter were clustered by genomes and families.

### 2.6. *Lvb. brevis* phenotype microarray analysis

Four autochthonous *Lvb. brevis* (14.2.10, 30.2.29, 7.8.43, and 3.2.41) and two allochthonous isolates, ATCC14869 (type strain) and ATCC367, were used for phenotype microarray (PM) analysis using the PM01 and PM02 plates of the Omnilog system (Biolog, Hayward, CA) following the manufacturer's instructions. Aliquots of 100 μl of the cell suspensions were added per well and plate. Duplicate PM01 and PM02 plates were inoculated with each isolate. The PM01 and PM02 plates were incubated at 33°C for 48 h in the Omnilog System, as recommended by the manufacturer.

The absorbance as a function of time data from the Omnilog System for each isolate can be found at https://doi.org/10.15482/USDA.ADC/1528683 (Page, 2023) and was used to calculate the growth rate using a Microsoft Excel spreadsheet and the following equation: = [LOG (End of Log Phase Time)-LOG (Start of Log Phase Time)] × 2.303)/(Absorbance at the End of Log Phase—Absorbance at the Start of Log Phase). The start and end of the log phase time and absorbance were adjusted for each PM plate. Averages and standard deviations were calculated for duplicate plates. Growth rate values for a given substrate that were above the standard deviation of the negative control value were considered utilized.

## 3. Results

### 3.1. Genotyping of the *Lvb. brevis* isolates using Rep-PCR-(GTG)_5_

**Figure 1:**
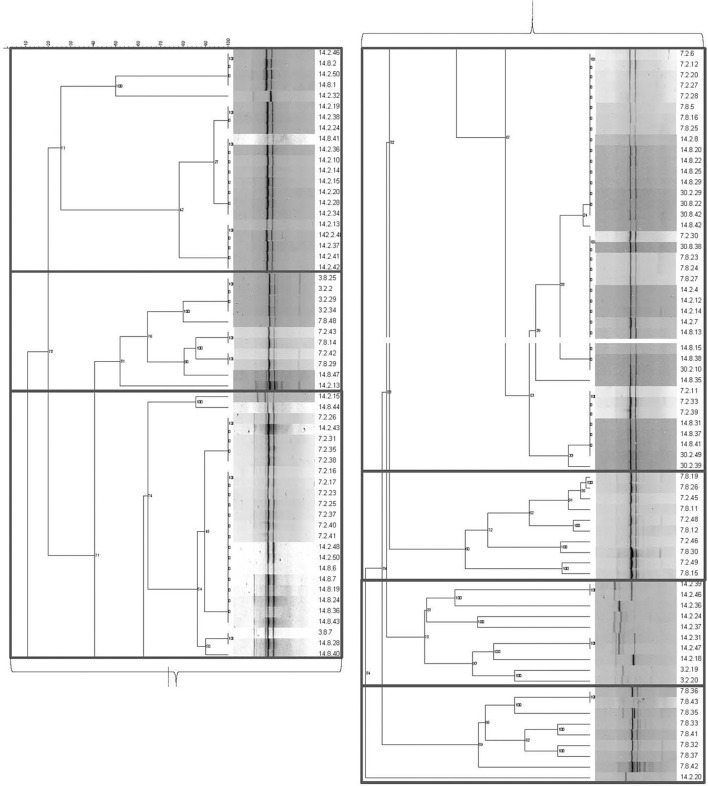
Genotyping dendrogram for autochthonous *Lvb. brevis*. Phylogenetic dendrogram and rep-PCR-(GTG)_5_ band pattern for 131 *Lvb. brevis* isolated from cucumber fermentation.

Figure 1 illustrates the seven genotypic clusters defined by the amplification of (GTG)_5_ repeats. The *Lvb. brevis* isolates produced two main bands from rep-PCR-(GTG)_5_, which are followed by one to two bands of higher or lower molecular weight (Figure 1).

### 3.2. Phenotyping of the *Lvb. brevis* isolates using a fermentation ability screening design

**Figure 2:**
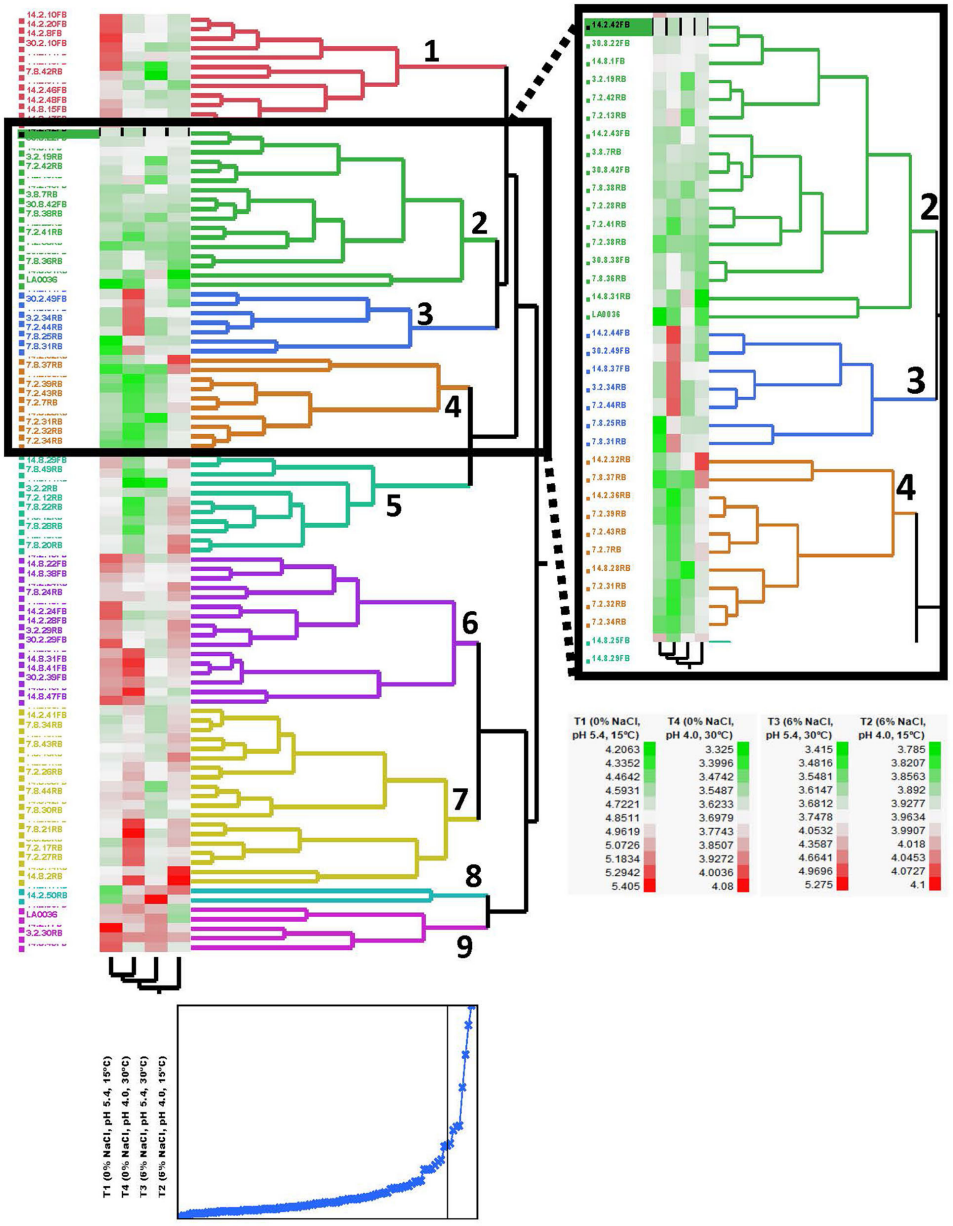
Phenotyping dendrogram for autochthonous *Lvb. brevis*. Hierarchical cluster analysis of *Levilactobacillus brevis* isolated from a commercial cucumber fermentation. Two-way clustering of cultures based on 48-h fermentation pH under varying conditions of salt, initial pH, and temperature. The plot below the hierarchical cluster analysis dendrogram shows the cumulative distance between clusters with decreasing cluster number, and the black vertical line marks the value for the 9 clusters shown in the dendrogram.

**Figure 3:**
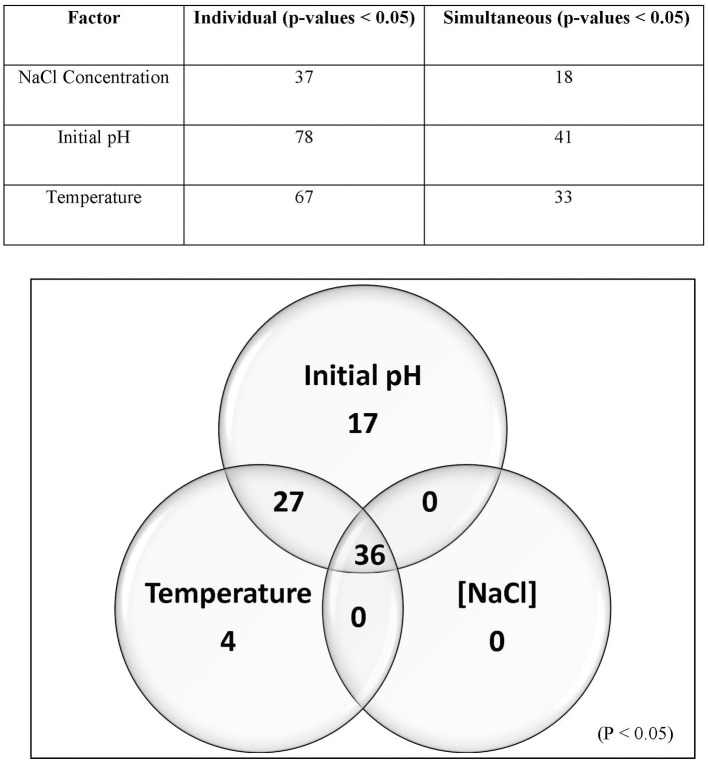
Screening analysis and Venn diagram illustrating the proportion of *Lvb. brevis* isolates affected by one or more fermentation conditions. Percentage (table) and number (Venn diagram) of isolates significantly affected by one or more fermentation variables (*n* = 97; *p-*value = 0.05).

The 99 Carolinian *Lvb. brevis* isolates acidified the treatment media, CJM, to a mean value of 4.86 ± 0.34 on treatment 1 and 3.96 ± 0.10 on treatment 2, both incubated at 15°C. Incubation at 30°C resulted in pH reductions to 3.77 ± 0.38 and 3.70 ± 0.25 after 2 days for treatments 3 and 4, respectively. Within these general trends, there was a wide range in performance among the isolates, which clustered into nine groups based on their ability to acidify CJM under varying conditions (Figure 2). The initial pH and fermentation temperature impacted the largest number of isolates, and there was no *Lvb. brevis* solely influenced by NaCl concentration (Figure 3). The 36 *Lvb. brevis* cultures that were impacted by NaCl concentration were also influenced by initial pH and/or temperature.

### 3.3. Comparative analysis of 14 *Lvb. brevis* genomes

**Figure 4:**
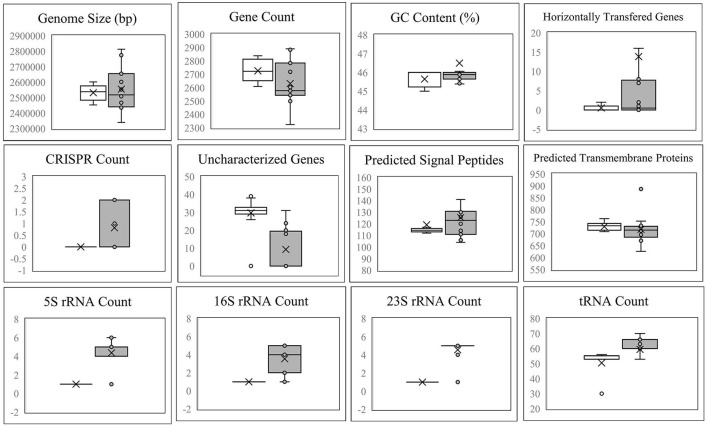
Descriptive data for the *Lvb. brevis* genome sequences. Box and whisker plot of the statistical data for the genomic DNA sequences derived from *Lvb. brevis* autochthonous to cucumber fermentation (gray box) relative to *Lvb. brevis* YSJ3, SA-C12, TMW1.2108, and NPS-QW-145 (white box).

**Figure 5:**
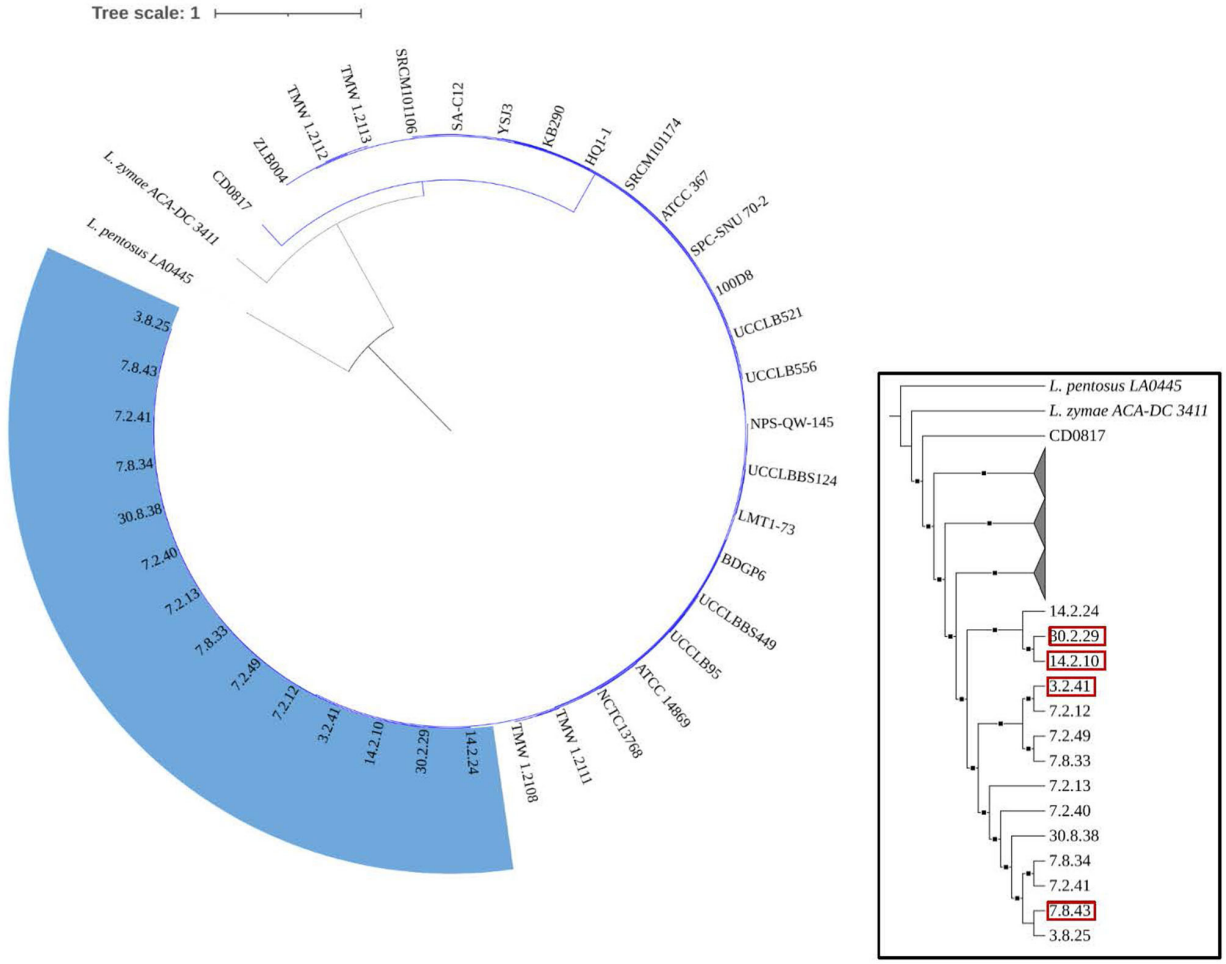
Phylogeny of the *Lvb. brevis* isolates relative to reference strains. Phylogenetic trees including autochthonous *Lvb. brevis* isolates sequenced (highlighted in blue) and reference strains constructed by aligning 477 common ORFs using the BVBRC Codon Tree. The trees were rooted with *Lpb. pentosus* LA0445. Isolate identifiers framed in red represent *Lvb. brevis* used in subsequent experiments. The inset tree is presented without scale and with clades including reference strains collapsed to better indicate relationships between autochthonous isolates. The branches marked with squares indicate a bootstrap value of 90 or greater.

Figure 4 demonstrates that the size, gene count, and GC content of the genome sequences derived from the 14 selected *Lvb. brevis* are within the expected ranges. Horizontal gene transfer, CRISPR loci, and ribosomal RNA clusters are more frequently detected in the cucumber fermentation autochthonous *Lvb. brevis* genomes relative to their reference allochthonous counterparts (Figure 4). The phylogenetic tree constructed with 477 open reading frames suggested that the autochthonous *Lvb. brevis* cluster among themselves and separate from allochthonous strains (Figure 5) isolated from kimchi, silage, wheat beer, and Yeshanjun style-fermented vegetables (Feyereisen et al., 2019). An ANI score matrix suggests that the autochthonous *Lvb. brevis* 7.8.43 is identical to the allochthonous strains included in the study scoring from 97.84 to 98.02, while the remaining thirteen autochthonous strains clustered apart with scores from 99.61 to 99.85 (Table 1).

**Table 1:** ANI score matrix comparing the *Lvb. brevis* isolates autochthonous to cucumber fermentation and reference strains SA-C12, YSJ3, TMW1.2, and NPS-QW-145.

	**14.2.24**	**3.2.41**	**3.8.25**	**30.8.38**	**7.2.12**	**7.2.13**	**7.2.40**	**7.2.41**	**7.2.49**	**7.8.33**	**7.8.34**	**7.8.43**	**SA-C12**	**YSJ3**	**NPS -QW- 145**	**TMW1.2108**
NPS-QW-145																97.34
YSJ3															97.26	97.25
SA-C12														97.56	97.38	97.29
7.8.43													97.91	97.49	97.45	97.27
7.8.34												97.97	97.87	97.55	97.38	97.30
7.8.33											99.70	97.94	97.89	97.50	97.44	97.11
7.2.49										99.75	99.71	97.90	97.87	97.53	97.42	97.20
7.2.41									99.85	99.84	99.80	98.06	98.01	97.44	97.38	97.29
7.2.40								99.72	99.69	99.62	99.57	97.83	97.79	97.47	97.40	96.89
7.2.13							99.76	99.75	99.75	99.75	99.74	98.02	97.96	97.51	97.41	97.19
7.2.12						99.81	99.76	99.73	99.72	99.70	99.69	97.91	97.83	97.51	97.37	97.09
30.8.38					99.79	99.76	99.74	99.71	99.71	99.68	99.67	98.00	97.90	97.55	97.32	97.14
3.8.25				99.82	99.80	99.77	99.74	99.72	99.71	99.70	99.70	97.84	97.82	97.52	97.39	97.16
3.2.41			99.85	99.84	99.83	99.83	99.80	99.73	99.72	99.69	99.64	97.96	97.92	97.50	97.35	97.10
14.2.24		99.80	99.77	99.75	99.75	99.73	99.69	99.68	99.67	99.64	99.62	97.93	97.91	97.47	97.25	97.07
14.2.10	99.81	99.80	99.80	99.78	99.76	99.75	99.69	99.67	99.66	99.63	99.61	97.97	97.88	97.47	97.30	97.15

### 3.4. Comparative analysis of the putative and deduced protein profiles and protein family sorting

Homogeneity of the putative proteins encoded by the *Lvb. brevis* genomes was observed regardless of origin (Supplementary Figure 1), with minor differences located at the end of contigs impacting a low molecular weight tyrosine phosphatase and ABC transporter, a permease protein, a transport protein, and several putative hypothetical proteins. However, the autochthonous *Lvb. brevis* lacks putative citrate lyase genes (Supplementary Table 2). The allochthonous and type strain *Lvb. brevis* ATCC14869, as well as other allochthonous strains, harbor putative citrate lyase genes in the vicinity of the NADP-dependent malic enzyme (Supplementary Figure 2). We also found that putative genes encoding for the varied elements of the 1,2-propanediol utilization (Pdu) microcompartment are conserved among *Lvb. brevis* strains and organized in a cluster (Supplementary Table 2).

The protein family sorting identified putative genes coding for malate, arginine, and glutamate utilization and L-/D-lactic acid production in all the genomes studied with a range of copy numbers (Supplementary Figure 3). However, a second copy of D-lactate dehydrogenase was only present in *Lvb. brevis* 7.8.43 and 3.2.41. Differences were observed in 13% of the putative coding genes, which were associated with mobile elements and hypothetical proteins (Supplementary Figure 3).

### 3.5. *Lvb. brevis* phenotype microarray analysis

**Figure 6:**
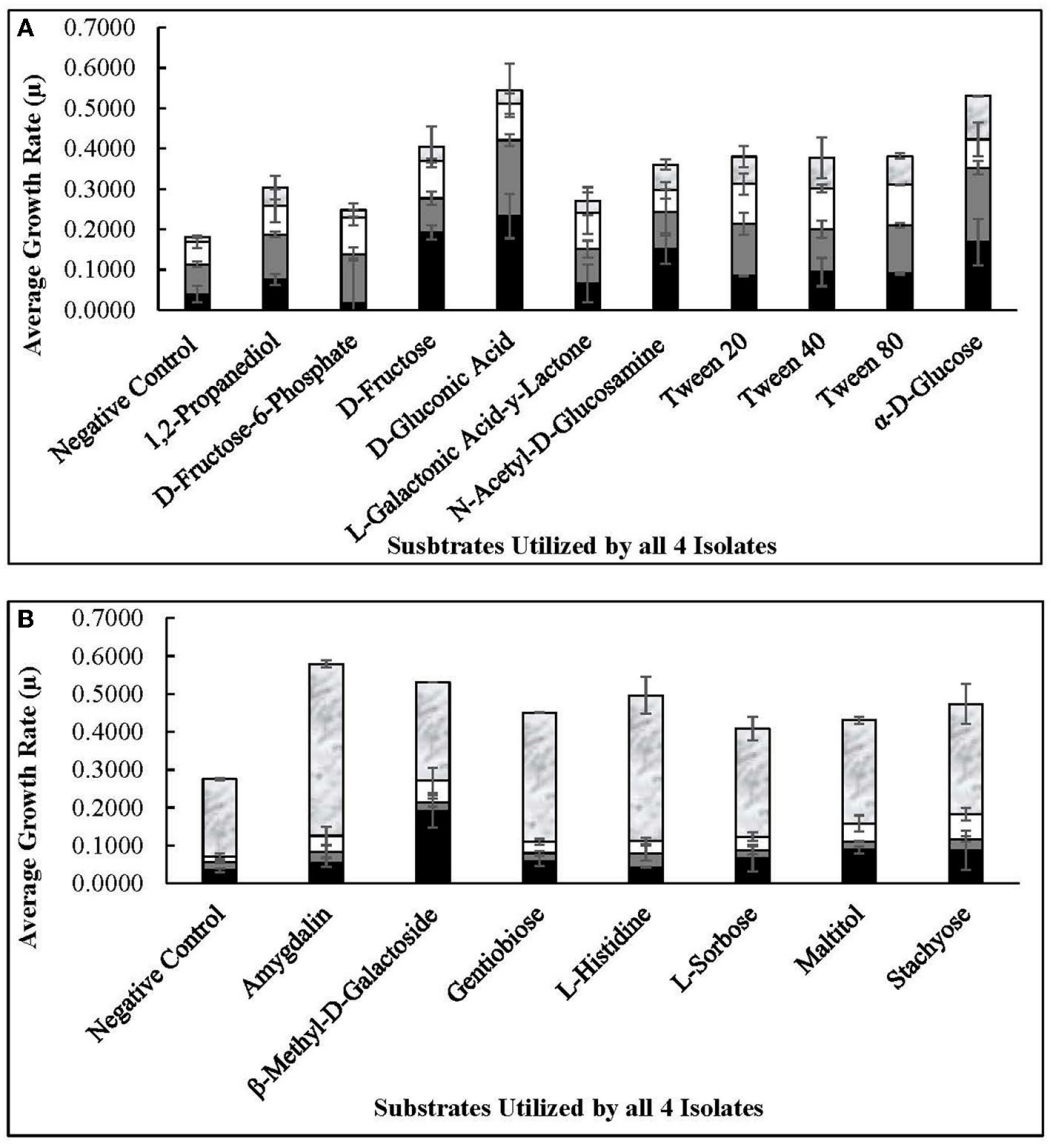
Commonly used substrates by *Lvb. brevis* isolates. Carbohydrates utilized by all four *Levilactobacillus brevis* isolates included in the comparative genomic analysis, including 7.8.43 (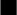), 14.2.10 (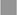), 30.2.29 (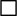), and 3.2.41 (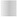), as determined by the PM01 **(A)** and PM02 **(B)** plates of the Omnilog for phenotype microarray.

**Figure 7:**
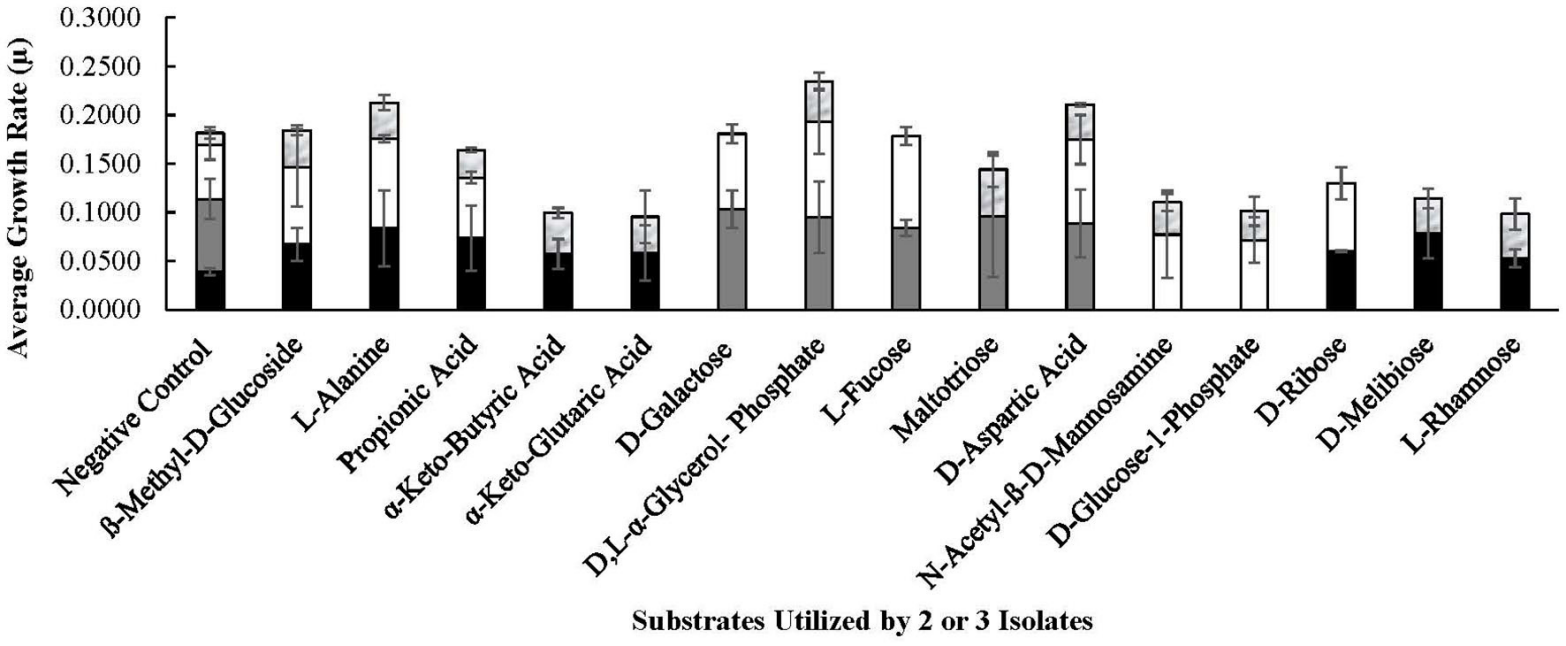
Substrates utilized by selected *Lvb. brevis*. Carbohydrates utilized by 2 or 3 *Levilactobacillus brevis* isolates included in the comparative genomic analysis, including 7.8.43 (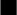), 14.2.10 (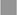), 30.2.29 (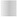), and 3.2.41 (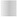), as determined by the PM01 and PM02 plates of the Omnilog for phenotype microarray.

The four autochthonous isolates, 7.8.43, 14.2.10, 30.2.29, and 3.2.41, utilized D-glucose, D-fructose, fructose-6-phosphate, L-sorbose, gluconic acid, and gentiobiose, among 13 other substrates (Figures 6, 7). While the allochthonous isolates, ATCC14869 and ATCC367, utilized xylose, trehalose, and cellobiose with growth rates ranging between 0.02005 and 0.04648, the autochthonous *Lvb. brevis* did not (Table 2). However, the autochthonous *Lvb. brevis* utilized gentiobiose, amygdalin, maltitol, and stachyose.

**Table 2:** Carbon sources utilized by single *Levilactobacillus brevis* as determined by the PM01 and P02 plates of the Omnilog.

**Substrate**	**Average growth rate (μ)**	**Standard deviation**	**Substrate**	**Average growth rate (μ)**	**Standard deviation**
**PM01 plate**			L-Asparagine	0.032	0.003
***Levilactobacillus brevis*** **7.8.43**			L-Lactic Acid	0.027	0.003
Negative control	0.039	0.004	L-Threonine	0.031	0.015
D-Glucose-6-phosphate	0.067	0.018	Sucrose	0.036	0.001
Pyruvic acid	0.073	0.044	α-D-Lactose	0.033	0.022
***Levilactobacillus brevis*** **14.2.10**			α-Methyl-D-Galactoside	0.042	0.001
Negative control	0.074	0.020	L-Alanyl-Glycine	0.046	0.011
2-deoxy adenosine	0.101	0.063	**PM02 Plate**		
***Levilactobacillus brevis*** **30.2.29**			***Levilactobacillus brevis*** **7.8.43**		
Negative control	0.055	0.015	Negative Control	0.035	0.005
D-Psicose	0.069	0.037	D-Tartaric Acid	0.042	0.006
L-Alanine	0.092	0.004	α-Methyl-D-Mannoside	0.127	0.008
L-Serine	0.077	0.010	***Levilactobacillus brevis*** **14.2.10**		
***Levilactobacillus brevis*** **3.2.41**			L-Proline	0.107	0.037
Negative control	0.013	0.006	***Levilactobacillus brevis*** **30.2.29**		
Acetoacetic acid	0.027	0.001	Negative Control	0.016	0.007
Adenosine	0.037	0.012	Xylitol	0.025	0.004
Adonitol	0.035	0.007	2,3-Butanedione	0.016	0.009
**D, L-malic acid**	0.032	0.012	3-Hydroxy 2-Butanone	0.020	0.010
D, L-α-glycerol-phosphate	0.041	0.009	Itaconic Acid	0.022	0.001
D-Cellobiose	0.028	0.001	Laminarin	0.025	0.007
D-Glucosaminic acid	0.031	0.016	L-Methionine	0.016	0.009
**D-malic acid**	0.035	0.019	***Levilactobacillus brevis*** **3.2.41**		
D-Mannose	0.074	0.001	Negative Control	0.204	0.00350
D-Sorbitol	0.037	0.009	i-Erythritol	0.228	0.02842
D-Threonine	0.032	0.008	Acetamide	0.258	0.00143
D-Trehalose	0.035	0.001	Maltose	0.047	0.0021
Formic acid	0.024	0.010	**ɤ-Amino Butyric Acid**	0.346	0.00765
Inosine	0.026	0.012	ɤ-Hydroxy Butyric Acid	0.230	0.03254
Glycerol	0.050	0.036			

As expected from the comparative analysis of putative proteins, only the allochthonous *Lvb. brevis* ATCC14869 was able to utilize citric acid in the PM01 plate, presenting a growth rate of 0.04521 ± 0.00828 in the presence of the substrate relative to 0.01617 ± 0.00458 from the negative control (Table 2). The diol, 1,2-propanediol, was utilized by the four autochthonous *Lvb. brevis* and the two allochthnous strains tested (Figure 6). Similarly, three out of the four autochthonous *Lvb. brevis* and *Lvb. brevis* ATCC14869 utilized propionic acid (Figure 7), and *Lvb. brevis* 30.2.29 and ATCC367 utilized sorbic acid in the PM02 plate with growth rates of 0.04613 ± 0.01385 and 0.02194 ± 0.00195, respectively. The surfactants, polysorbate 20, 40, and 80 were utilized by all autochthonous *Lvb. brevis* (Figure 6). Figures 6, 7 also show that the autochthonous *Lvb. brevis* utilizes amino sugars, such as N-acetyl-D-glucosamine and N-acetyl-D-mannosamide, and glucosides, such as β-methyl-D-glucoside and β-methyl-D-galactoside. Table 2 shows that some autochthonous *Lvb. brevis* utilize an assortment of amino acids, nucleotides, and organic acids. *Lvb. brevis* 3.2.41 utilizes γ-amino butyric acid.

## 4. Discussion

Rep-PCR-(GTG)_5_ is considered the technique with the best discrimination power for intraspecies biodiversity among lactic acid bacteria (Gevers et al., 2001; Kostinek et al., 2005; Anekella and Pérez-Díaz, 2020). Contrary to *Lpb. plantarum* and *Lpb. pentosus*, the *Lvb. brevis* rep-PCR-(GTG)_5_ patterns suggest limited genetic diversity (Panagou et al., 2008; Pino et al., 2018; Pérez-Díaz et al., 2021), which agrees with reports made by Tamang et al. (2005) for varied lactobacilli. *Lpb. plantarum* is known for its nomadic lifestyle, and its genome reflects diversity and plasticity resulting from its ability to adapt to various habitats (Martino et al., 2016). *Lvb. brevis*, however, presents a prolonged doubling time in cucumber fermentation (Pérez-Díaz et al., 2017) and is associated with specific niches, such as the human microbiota and spoilage of fermented foods such as beer (Feyereisen et al., 2019).

Seven and nine clusters were identified in the dendrograms generated with the results of the rep-PCR-(GTG)_5_ and the fermentation ability assay, respectively, with divergent isolates distribution (Figures 1, 2). The pH data from the fermentation ability assay indicated an enhancement in acid production by *Lvb. brevis* at the higher temperature (30°C) in CJM. Figure 2 indicates that most of the *Lvb. brevis* cultures were able to perform well under theoretically optimal conditions of 6% NaCl, initial pH 5.4, and 30°C as evidenced by the primarily green third column from the left in the Hierarchical Cluster Analysis dendrogram. The most robust isolates for adjunct cultures were grouped together in clusters 2 and 4, which were comprised of 36 *Lvb. brevis* cultures that demonstrated the ability to acidify CJM across all conditions (Figure 2). The fermentation ability data also suggest that potential synergies between the three variables tested were more useful in screening for robust adjunct culture candidates. It is inferred that the lack of performance of selected *Lvb. brevis* under sub-optimal conditions responds to their inability to adapt to multiple stresses, population densities, and/or growth conditions.

Understanding the divergence in the clustering derived from rep-PCR-(GTG)_5_ and the fermentation ability assay, we selected fourteen isolates that represent each of the clusters for whole genome sequencing. The availability of such genome sequences in public databases was announced by Page et al. (2022). Differences in the incidence of CRISPR loci and horizontal gene transfer in favor of the *Lvb. brevis* strains reinforce the concept of microbial adaptation to habitats (Martino et al., 2016). The number of uncharacterized genes is significantly lower in the autochthonous *Lvb. brevis* genomes as compared to the allochthonous strains (Figure 4), suggesting the need for specialization in cucumber fermentations is relatively lower. ANI scores suggest that differences at the genome level exist among *Lvb. brevis* isolated from varied fermentation habitats and that one strain prevails in cucumber fermentation.

Contrary to the allochthonous *Lvb. brevis* ATCC 14869, the autochthonous strain was found to be unable to metabolize citric acid. While citric acid is central to the tricarboxylic acid cycle and aerobic respiration, it is absent in immature cucumbers (McFeeters et al., 1982). Lactic acid bacteria can use it to generate alternative products such as acetate, ethanol, lactate, succinate, and acetoin in exchange for a proton motive force or ATP production by substrate-level phosphorylation (Gänzle, 2015). The allochthonous *Lvb. brevis* ATCC14869 strain produces the characteristic aroma associated with diacetyl production on the testing medium and CJM (data not shown). Further studies are needed to determine whether the absence of citrate lyase in the autochthonous *Lvb. brevis* is detrimental to competitive growth as a starter culture.

The autochthonous *Lvb. brevis* utilized sugars associated with plant systems such as cereals, vegetables, and fruits (Buckenhüskes, 1993, 1997; Jones et al., 1999; Dumville and Fry, 2003; Bolarinwa et al., 2014; Lei et al., 2022) (Figure 6), as well as gentiobiose, a disaccharide intrinsic to cucumber (Ucar et al., 2020a). Fructose, glucose, xylose, and trehalose are present in cucumber fermentation (Ucar et al., 2020a,b), and their concentrations decline prior to or during spoilage led by *Lentilactobacillus buchneri* (Johanningsmeier and McFeeters, 2015). Fructose and glucose are preferably removed by lactobacilli in cucumber fermentation, including *Lvb. brevis*. Together, these observations suggest that a mixed culture of autochthonous and allochthonous *Lvb. brevis* may aid in preventing spoilage by removing not only gentiobiose but also trehalose and xylose post-primary fermentation.

**Figure 8:**
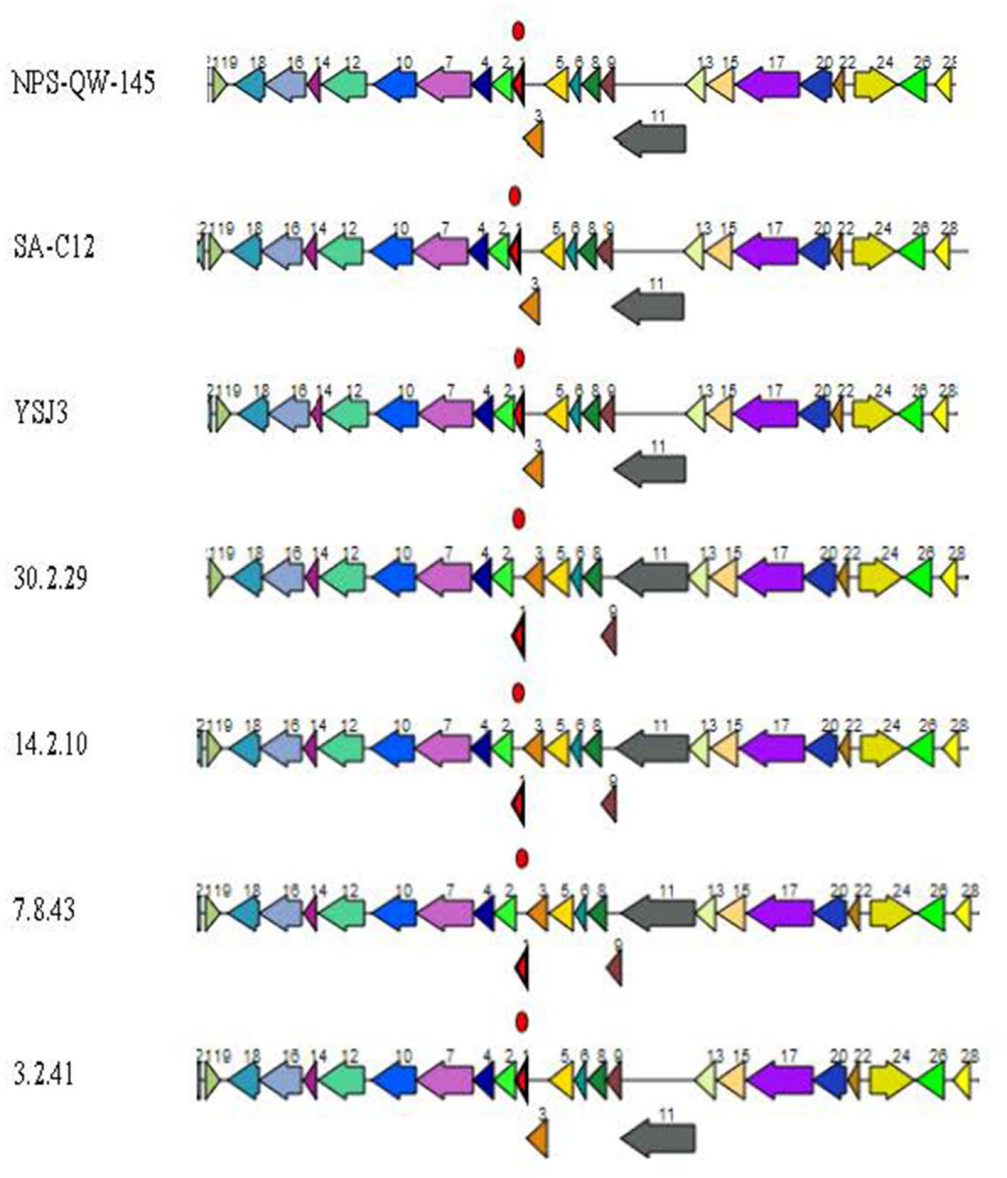
*Lvb. brevis* 1,2-propanediol utilization putative operon. Putative 1,2-propanediol utilization operon is found in allochthonous and autochthonous *Levilactobacillus brevis* isolates. Coding genes are (1) *pduN* polyhedral body protein, (2) *pduO* ATP:Cob**(I)**alamin adenosyltransferase, (3) *pduM* polyhedral body protein, (4) hypothetical protein, (5) *pduL* phosphate propanoyl transferase, (6) *pduJ* polyhedral body protein, (7) *pduO* CoA-acylating propionaldehyde dehydrogenase, (8) *pduK* polyhedral body protein, (9) *pduG* propanediol dehydratase reactivation factors small subunit, (10) *pduQ* propanol dehydrogenase, (11) *pduH* propanediol dehydratase reactivation factor large subunit, (12) *pduW* propionate kinase, (13) *pduC* propanediol dehydratase small subunit, (14) *pduU* polyhedral body protein, (15) *pduD* propanediol dehydratase medium subunit, (16) NADH:flavin oxidoreductase, (17) *pduE* propanediol dehydratase large subunit, (18) hypothetical protein, (19) phage transcriptional repressor, (20) *pduB* polyhedral body protein, (22) *pduA* polyhedral body protein, (24) propanediol utilization transcriptional activator, (26) propanediol diffusion facilitator, and (28) *pduV* propanediol utilization protein.

The identification of putative genes coding for the multiple components of Pdu microcompartments in all the *Lvb. brevis* genomes agrees with the observed ability of the species to utilize the substrates in the phenotyping assay using the Omnilog (Figure 8). 1-2-propanediol and propionic acid are produced in cucumber fermentation spoilage by *Le. buchneri* (Stefanie et al., 2001; Johanningsmeier and McFeeters, 2013). The diol is converted to propionaldehyde *via* a coenzyme B_12_-dependent diol dehydratase in Pdu microcompartments, which in turn is catabolized to 1-propanol and propionic acid (Liu et al., 2007). Such a pathway generates ATP, regenerates NAD + H^+^, and produces propionyl-CoA for central metabolism (Liu et al., 2007). The observations that all *Lvb. brevis* tested could metabolize 1,2-propanediol by expressing the putative Pdu microcompartment cluster suggest that such a compound may be key to the ability of the bacterium to colonize. Further studies are needed to define the conditions needed for *Lvb. brevis* to utilize the diol and to understand the advantages associated with its utilization within the fermentation habitat.

It is relevant to mention that *Lvb. brevis* isolates capable of utilizing the antifungal preservative sorbic acid, such as 30.2.29 and ATCC367, have limited application as starter cultures in industrial cucumber fermentation. Sorbic acid has been used for decades in pickling in the United States as a processing aid to control the undesired growth of yeast and molds, particularly on the surface of brines contained in open-top tanks (Etchells et al., 1961). The preservative has been proven useful in enabling industrial cucumber fermentation without sodium chloride (Pérez-Díaz et al., 2022). The effectiveness of the preservative is limited during long-term storage by its removal, presumably by microbial activity. Thus, starter cultures capable of utilizing the preservatives would be detrimental to the long-term stability of cucumber fermentations brined with low salt.

Our data suggest that polysorbates typically added to food products could positively impact the growth of the autochthonous *Lvb. brevis* (Figure 6). The mechanism by which such an effect is exerted and its relevance to vegetable fermentation need further studies. However, an enhancement of biofilm formation by *Lvb. brevis* and other lactobacilli in the presence of the surfactants could augment the probiotic content of fermented vegetable products, as in fermented olives (Benítez-Cabello et al., 2020). It is understood that the surfactants and/or deriving degradation products can influence bacterial growth and the ability to form biofilm in various ways (Nielsen et al., 2016).

The ability of *Lvb. brevis* to utilize amino sugars led us to inquire if *Lvb. brevis* can degrade amino sugars derived from cucumber tissue during long-term storage of the fermented fruit, as observed for the non-starter lactic acid bacterium, *Lactobacillus wasatchensis*, in aged Cheddar cheese (Lauret et al., 1996; Oberg et al., 1996; Plumbridge and Vimr, 1999; Uehara and Park, 2004). On the other hand, the polymeric pectin, which is also a component of plant cell walls, was only utilized by *Lvb. brevis* 14.2.10 in the PM02 plate. Because pectin degradation is associated with fermented cucumber tissue softening (Walter et al., 1985; McFeeters, 1992), this isolate would have limited application as a starter culture for vegetable fermentation.

Interestingly, *Lvb. brevis* 3.2.41 utilizes *gamma*-amino butyric acid, which is formed during indigenous cucumber fermentation (Fideler Moore, 2021) and may contribute to its health-promoting properties. Therefore, *Lvb. brevis* 3.2.41 would need further evaluation prior to use as an adjunct culture.

## 5. Conclusion

A genetically distinct *Lvb. brevis* prevails in cucumber fermentation, robustly utilizes glucose and fructose under varied conditions of pH, temperature, and salt, and presents distinct metabolic activities as compared to allochthonous strains. Such an autochthonous *Lvb. brevis* strain was isolated from two distinct, geographically distant fermentations and is genotypically defined by the absence of genes that encode for citrate lyase. We postulate that mixed cultures of allochthonous and autochthonous *Lvb. brevis* may outcompete spoilage microbes by utilizing key sugars intrinsic to cucumber. Members of the species encode for a complete and putative Pdu microcompartment cluster that theoretically enables the production of energy in significant ways.

## Data availability statement

The datasets presented in this study can be found in online repositories. The names of the repository/repositories and accession number(s) can be found in the article/Supplementary material.

## Author contributions

IP-D contributed to the scientific approach, experimental design, data generation and interpretation, comparative proteomic and genomics analyses, supervision, project management, and the writing and editing of the manuscript. LM-S executed the Rep-PCR-(GTG)_5_ analysis, the corresponding data processing and reviewed the manuscript. SJ contributed the data analysis for the fermentation ability assay and Venn diagram and edited the manuscript. All authors contributed to the article and approved the submitted version.
