# Supplementary material for: Levilactobacillus brevis, autochthonous to cucumber fermentation, is unable to utilize citric acid and encodes for a putative 1,2-propanediol utilization microcompartment
Source: Front Microbiol. 2023 Jul 26;14:1210190. doi: 10.3389/fmicb.2023.1210190 (PMC10410858; doi:10.3389/fmicb.2023.1210190)
Supplement: Supplementary file 1 [file Table_1.DOCX]

**Supplementary Table 1:** Record locators for the 14 *Lvb. brevis* genomes used in this study.

| Isolate Identifier | GenBank Accession Number | SRA Accession Number |
| --- | --- | --- |
| 14.2.10 | [JAGXCC000000000](https://identifiers.org/resolve?query=insdc:JAGXCC000000000) | [SRR14682955](https://identifiers.org/resolve?query=insdc.sra:SRR14682955) |
| 14.2.24 | [JAGXCD000000000](https://identifiers.org/resolve?query=insdc:JAGXCC000000000) | [SRR1468295](https://identifiers.org/resolve?query=insdc.sra:SRR14682955)4 |
| 3.2.41 | [JAGXIY000000000](https://identifiers.org/resolve?query=insdc:JAGXIY000000000) | [SRR146829](https://identifiers.org/resolve?query=insdc.sra:SRR14682955)23 |
| 3.8.25 | JAJHNO000000000 | SRR16612720 |
| 7.2.12 | JAJHOC000000000 | [SRR16675615](https://dataview.ncbi.nlm.nih.gov/object/SRR16675615) |
| 7.2.40 | JAJHVM000000000 | [SRR16675614](https://dataview.ncbi.nlm.nih.gov/object/SRR16675614) |
| 7.2.41 | JAJHVN000000000 | [SRR16675613](https://dataview.ncbi.nlm.nih.gov/object/SRR16675613) |
| 7.2.49 | JAJHVO000000000 | [SRR16675612](https://dataview.ncbi.nlm.nih.gov/object/SRR16675612) |
| 7.2.13 | JAJHVP000000000 | [SRR16675611](https://dataview.ncbi.nlm.nih.gov/object/SRR16675611) |
| 7.8.33 | JAJHVQ000000000 | [SRR16675610](https://dataview.ncbi.nlm.nih.gov/object/SRR16675610) |
| 7.8.34 | JAJHVR000000000 | [SRR16675609](https://dataview.ncbi.nlm.nih.gov/object/SRR16675609) |
| 7.8.43 | JAJHVS000000000 | [SRR16675608](https://dataview.ncbi.nlm.nih.gov/object/SRR16675608) |
| 30.2.29 | JAJHVT000000000 | [SRR16675605](https://dataview.ncbi.nlm.nih.gov/object/SRR16675605) |
| 30.8.38 | JAJHVU000000000 | [SRR16675616](https://dataview.ncbi.nlm.nih.gov/object/SRR16675616) |
| ATCC 14869 | AWVK00000000 | SRR1151178 |
| SA-C12 | CP031185.1 | Not Available |
| YSJ3 | CP092264.1 | Not Available |
| NPS-QW-145 | CPO15398.1 | Not Available |
| TMW1.2108 | CP019737.1 | Not Available |
