# Supplementary material for: Levilactobacillus brevis, autochthonous to cucumber fermentation, is unable to utilize citric acid and encodes for a putative 1,2-propanediol utilization microcompartment
Source: Front Microbiol. 2023 Jul 26;14:1210190. doi: 10.3389/fmicb.2023.1210190 (PMC10410858; doi:10.3389/fmicb.2023.1210190)

Supplementary Figure 1: *Lvb. brevis* genome comparative analysis. Deduced protein sequence-based genome comparison of *Lvb. brevis* reference strains and isolates using bidirectional BLASTP. *Lvb. brevis* isolates shown are, from the outer track to the inner one, ATCC14869, 14.2.10, 7.8.43, 3.2.41, 30.2.29, SA-C12, YSJ3, and NPS-QW-145. The percent protein sequence identity is color coded, where dark blue is 100, light blue is 99, light green is 95, yellow is 80, orange is 60, and red is 20 all applicable to the bidirectional best hit.

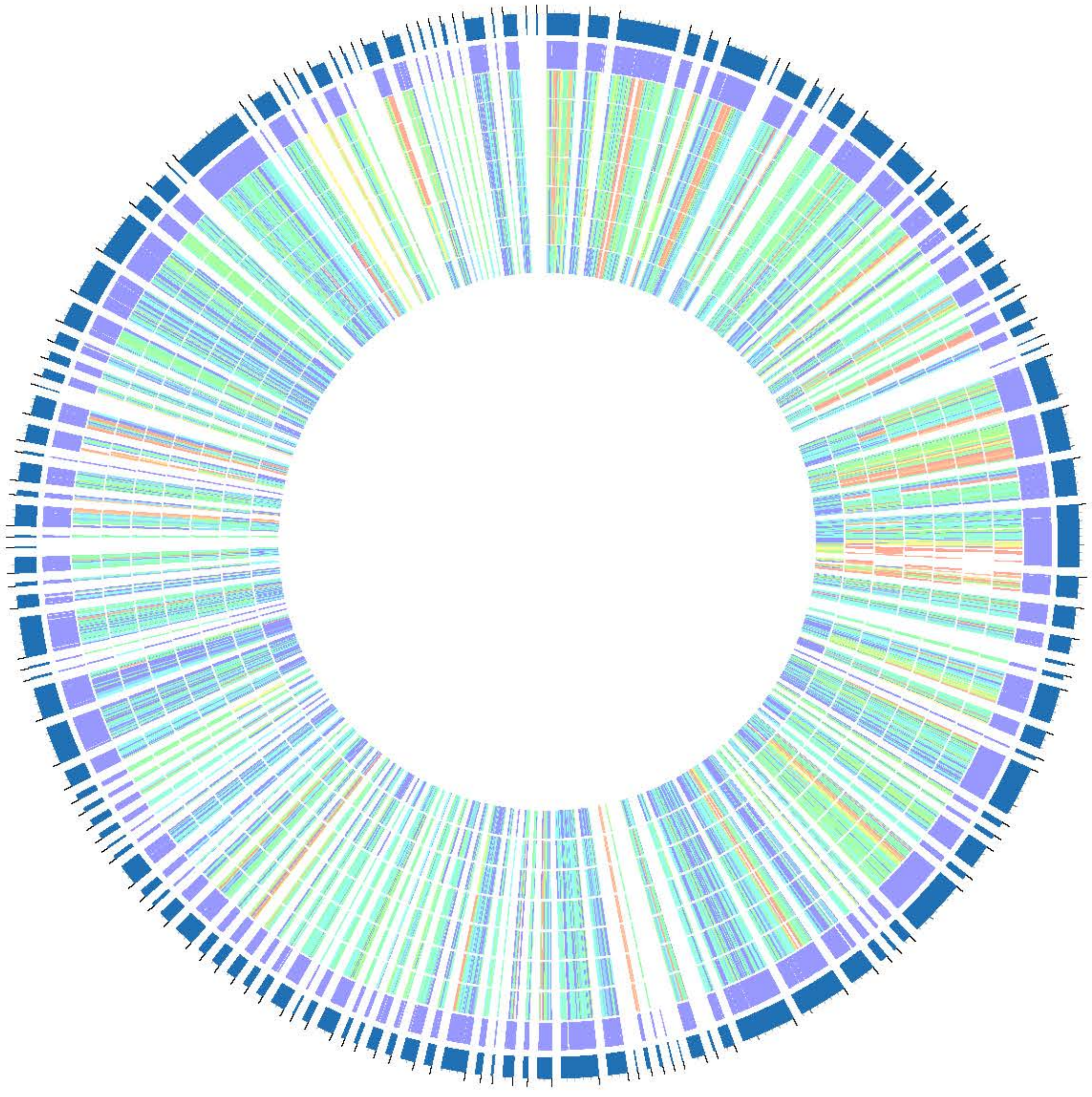

Supplementary Figure 2: Alignments of the citrate lyase coding region in *Lvb. brevis* isolates and reference strains. Output of the Compare Region Viewer using PATRIC cross-genus families (PGfams) for the reference genome *Lvb. brevis* SA-C12 and representative matching genomes, allochthonous *Lvb. brevis* in this case.

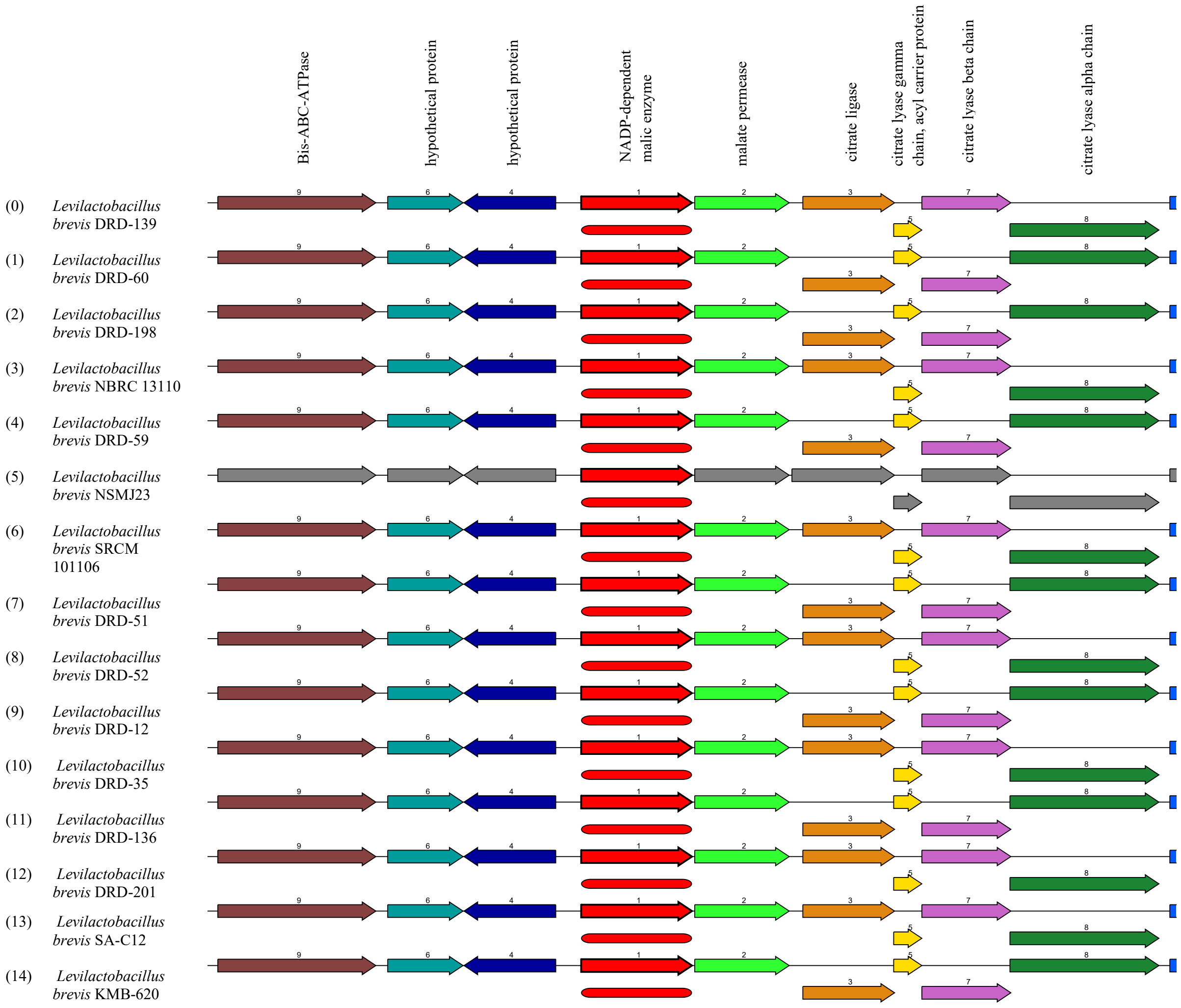

Supplementary Figure 3: *Lvb. brevis* putative protein family sorting. Sorting of the deduced protein family homologs in *Lvb. brevis* reference strains and experimental isolates clustered by genomes and families (a). Expanded view of the sorting for malate, arginine and glutamate utilization, D- and L-lactate dehydrogenases, transposition elements, and hypothetical proteins (b). Deduced protein sequences found with a copy number of 0, 1, 2 or 3 and more are identified by the black, light yellow, dark yellow and pink, respectively.

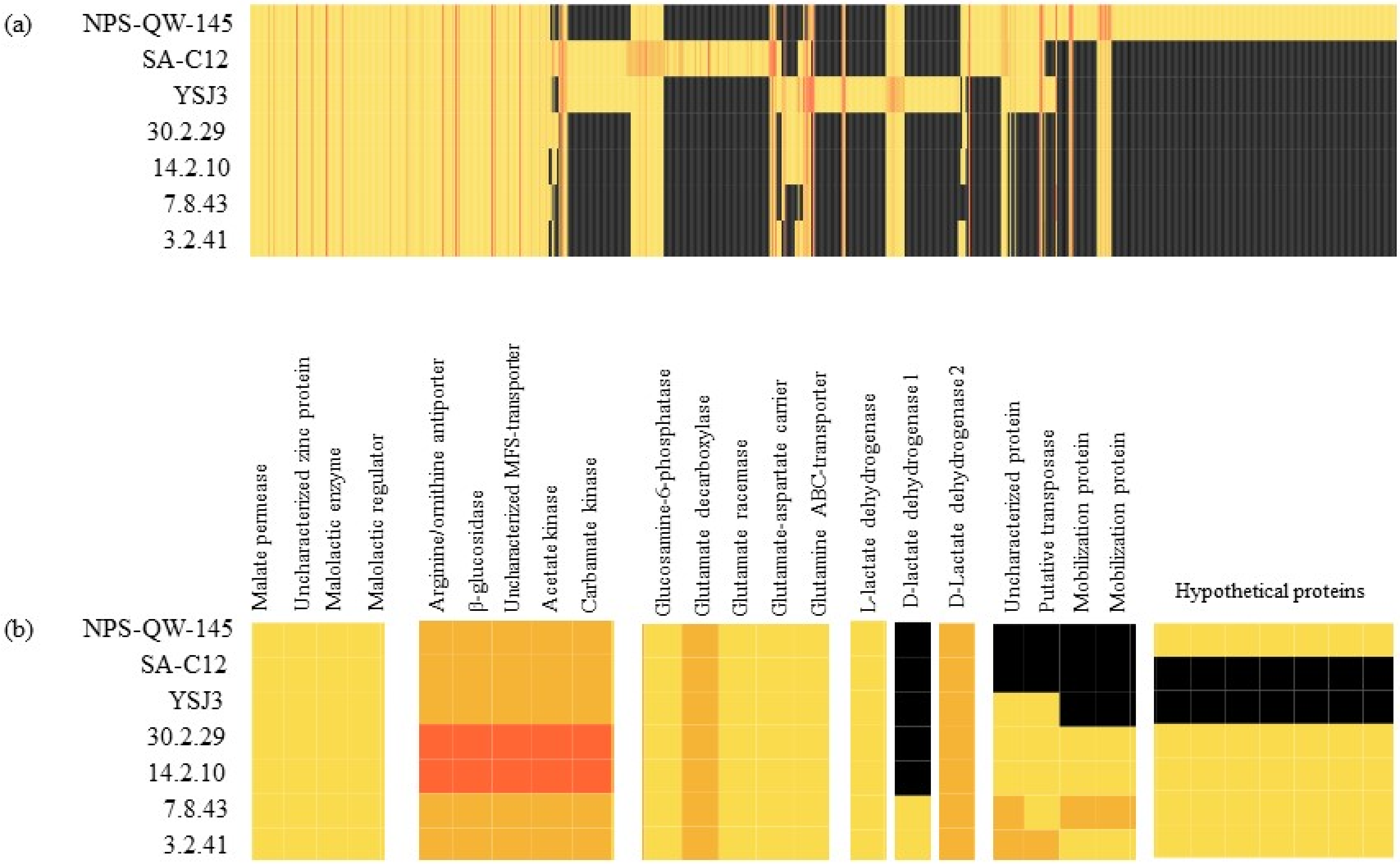

Supplement: Supplementary file 3 [file Data_Sheet_1.pdf]
